# Short-term health impacts of air pollution in Canada: a nonlinear multi-pollutant Bayesian case-crossover framework

**DOI:** 10.1093/ije/dyag194

**Published:** 2026-09-22

**Authors:** Guowen Huang, Patrick E Brown, Hwashin Hyun Shin

**Affiliations:** Department of Statistical and Actuarial Sciences, Western University, London, Ontario, Canada; Centre for Global Health Research, St Michael’s Hospital, Toronto, Ontario, Canada; Department of Statistical Sciences, University of Toronto, Toronto, Ontario, Canada; Environmental Health Science and Research Bureau, Health Canada, Ottawa, Ontario, Canada; Department of Mathematics and Statistics, Queen’s University, Kingston, Ontario, Canada

**Keywords:** air pollution, case-crossover, Bayesian hierarchical model, nonlinear exposure-response, multi-pollutant analysis

## Abstract

**Background:**

Most short-term air pollution studies rely on linear, single-pollutant models that cannot adequately describe the nonlinear and interdependent nature of pollutant-health associations.

**Methods:**

We developed a nonlinear multi-pollutant Bayesian case-crossover model to jointly evaluate short-term health effects of PM${\;}_{2.5}$, NO_2_, and O_3_. The model accommodates overdispersion, propagates exposure uncertainty from imputed monitoring data, and pools Census Division (CD)-specific exposure-response functions through a Bayesian hierarchical framework. We applied this approach to cause-specific daily mortality (2001–2015) and hospitalization (2001–2018) data across 20 Canadian CDs, covering more than half of the national population.

**Results:**

The estimated exposure-response relationships varied by pollutant and health outcome. The combined PM${\;}_{2.5}$+NO_2_ metric showed the clearest positive associations with all non-accidental mortality and hospitalization. Respiratory hospitalization estimates were higher among seniors, but evidence of an age difference remained uncertain. An approximate 10-unit contrast in combined PM${\;}_{2.5}$+NO_2_ corresponded to an estimated 1.5% (95% credible interval [CrI]: 0.3, 2.2) increase in all non-accidental mortality and a 0.6% (95% CrI: 0.0, 1.3) increase in all non-accidental hospitalization, while the corresponding estimate for senior respiratory hospitalization was 2.7% (95% CrI: 0.9, 4.5). Several PM${\;}_{2.5}$ and NO_2_ curves were nonlinear, whereas O_3_ associations were closer to linear over much of the observed range.

**Conclusion:**

Our study introduces a flexible epidemiologic modelling framework for quantifying the short-term health impacts of correlated pollutants. The results highlight the value of accounting for nonlinear and joint exposure patterns in multi-pollutant settings, while uncertainty remains substantial for several cause- and age-specific estimates.

Key Messages

- We evaluated short-term health effects of multiple air pollutants in Canada using a nonlinear multi-pollutant case-crossover framework.
- The combined PM_2.5_+NO_2_ metric showed the clearest estimated patterns for all non-accidental mortality and hospitalization, while several cause- and age-specific estimates remained uncertain.
- The framework provides a way to examine nonlinear and joint exposure patterns, but strong pollutant correlation and estimate uncertainty limit interpretation.

## Introduction

Air pollution remains a major global health concern, responsible for an estimated 4.2 million premature deaths annually [1]. Short-term increases in ambient pollutants have been consistently linked with excess daily mortality and hospitalization, particularly for cardiovascular and respiratory causes [2–8]. Many short-term epidemiologic analyses still consider pollutants separately or impose linear effects [7–11], even though real-world exposures occur as correlated mixtures. Increasing evidence suggests that associations are often nonlinear [12], with steeper effects at lower concentrations. Multi-pollutant modelling is therefore increasingly important in air-pollution epidemiology.

We address these challenges by proposing a nonlinear multi-pollutant Bayesian case-crossover framework that simultaneously models PM${\;}_{2.5}$, NO_2_, and O_3_ while accounting for uncertainty in imputed exposure series and overdispersion in day-to-day variability. The time-stratified case-crossover design compares each event day with matched referent days within the same temporal stratum, thereby controlling for time-invariant individual factors and seasonal patterns [4, 13, 14]. Embedding this design within a Bayesian hierarchical structure allows flexible estimation of pollutant-specific exposure-response functions and synthesis of CD-specific estimates into coherent national effects. Using daily mortality (2001–2015) and hospitalization (2001–2018) data from 20 Canadian CDs, we estimate nonlinear exposure-response relationships for multiple pollutants and assess their combined short-term health effects by age group and disease category.

## Methods

### Study region

**Figure 1:**
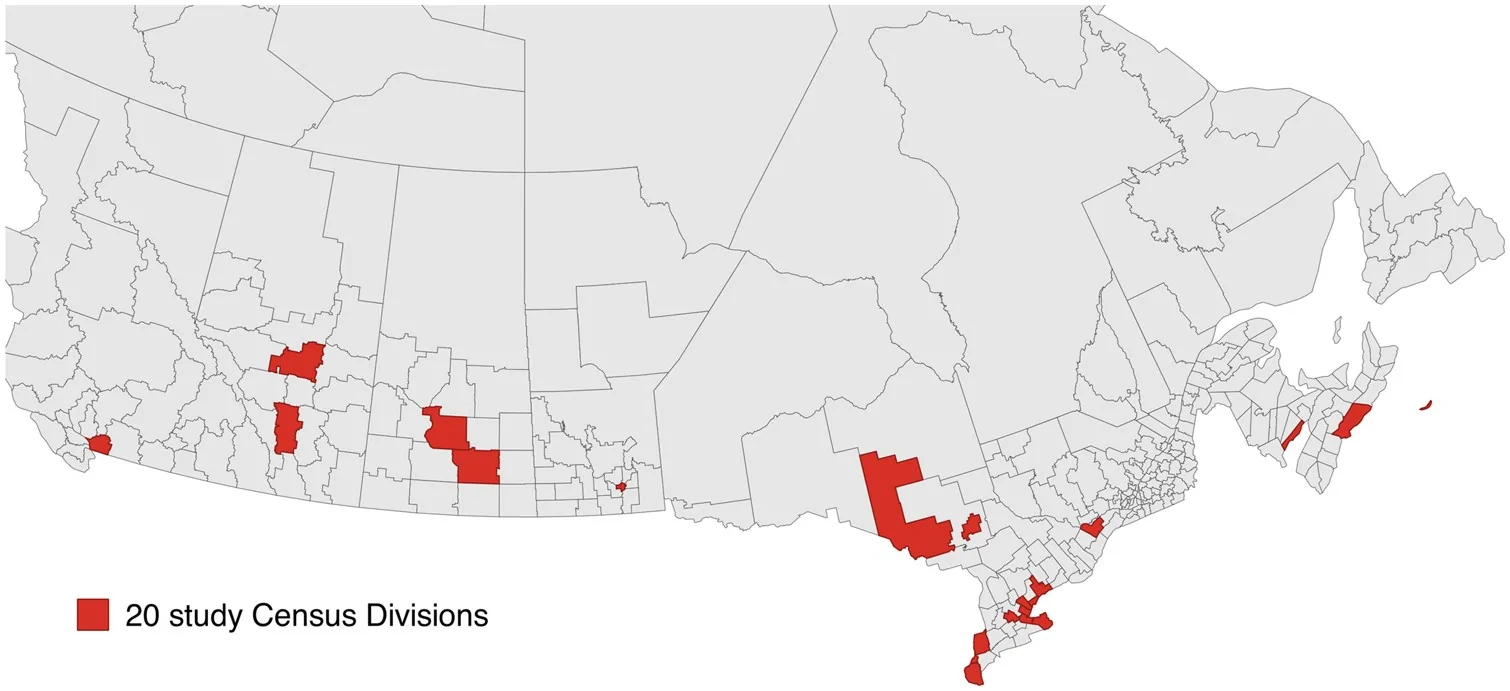
The 20 Census Divisions (in red) that comprise the study area.

The present research selected 20 Census Divisions (CDs) situated in 7 provinces throughout Canada, as shown by Fig. 1. Selection of these CDs was guided by the need to ensure high quality exposure estimation, which includes only CDs with at least two long term ground-monitoring stations providing 20 or more years of data prior to 2019. The 2016 population of the 20 CDs ranged from 74 020 (Saint John) to 2 731  571 (Toronto).

### Health outcome data

The study analyzed two sets of health outcome data: mortality records from 2001 to 2015 and hospitalization records from 2001 to 2018. The data used in this study is the most up-to-date available. Daily counts of mortality were obtained from the Canadian Vital Statistics Death Database managed by Statistics Canada, and hospitalization data were obtained from the Canadian Institute for Health Information (source: Hospital Morbidity Database and Discharge Abstract Database). Each dataset had details on age and cause of death or hospitalization, categorized according to the International Classification of Diseases, 10th Revision (ICD-10). Three age groups were considered: all-age, non-seniors aged 1 to 65, and seniors aged 66 and above. Three broad causes of non-accidental health outcomes were considered: all non-accidental (ICD-10 A00-R99), circulatory diseases (I00-I99), and respiratory diseases (J00-J99).

Table 1 presents an overview of total cases for mortality and hospitalization by age-group during the study period, while Supplementary Table 1 provides the same summaries for each CD. For mortality, there were 1 140  053 all non-accidental cases, with most occurring among seniors (899 586 vs. 240 467, or 79% vs. 21%). In contrast, the total number of all non-accidental hospitalizations was 14 875 768, with fewer cases among seniors than non-seniors (5 328 245 vs. 9 547 523, or 36% vs. 64%). Table 1 also summarizes the proportion of days with no reported cases (zero-case days). Mortality had higher zero-case proportions than hospitalization, especially for respiratory outcomes and for non-seniors. For hospitalization, zero-case proportions were small overall, indicating more stable daily counts across causes and age groups.

**Table 1:** Total cases for mortality (2001–2015) and hospitalization (2001–2018) by outcome and age group in 20 Canadian Census Divisions, with non-seniors aged 1 to 65 years and seniors aged 66 years and older.

	**Mortality**			**Hospitalization**		
Outcome	All-age	Senior	Non-senior	All-age	Senior	Non-senior
**Cases, n**						
All non-accidental	1 140 053	899 586	240 467	14 875 768	5 328 245	9 547 523
Circulatory	366 962	314 156	52 806	2 134 623	1 357 810	776 813
Respiratory	100 945	90 264	10 681	1 306 464	663 917	642 547
**Zero-case days, %**						
All non-accidental	1.32	2.43	22.21	0.00	0.01	0.00
Circulatory	11.47	14.73	62.65	0.45	1.47	5.67
Respiratory	46.50	49.92	90.02	1.79	7.07	8.56

*Notes:* Values in the first section are total numbers of cases; values in the second section are percentages of study days with zero reported cases.

### Exposure estimation

We analyzed PM${\;}_{2.5}$, NO_2_, and O_3_ using hourly observations from the National Air Pollution Surveillance (NAPS) Program [15]. To address missing data and outliers, we applied the Bayesian pollution model of [16], which borrows strength across pollutants, seasons, and related monitoring information. We generated 50 posterior predictive samples of hourly concentrations for each CD, aggregated them to daily exposures (24-hour mean for PM${\;}_{2.5}$ and NO_2_; 8-hour maximum for O_3_), and propagated this uncertainty due to missing hourly monitoring data through all subsequent health-effect analyses. Missingness was handled at the station-hour level, so uncertainty increased with missing monitoring values. Missingness also varied by pollutant, location, season, and calendar time because stations entered and exited service over the study period. Additional details are given in [16] and its supplementary material. In addition to air pollution, daily mean temperature was obtained from the National Climate Data and Information Archive of Environment and Climate Change Canada. For CDs with multiple weather stations, we used the within-CD average across available stations. Temperature was generally observed more completely than the pollutant exposures, but it is still represented here as a CD-day average and therefore may not fully capture within-CD spatial variation.

CD-specific summary statistics are provided in Supplementary Table 3. Because exposures were averaged to the CD-day level, estimates should be interpreted as area-level ambient exposure metrics and may be affected by spatial aggregation error, especially in larger CDs.

### Exposure-response model

To better capture the complex health effects of ambient air pollution, especially potential threshold effects and pollutant interactions, we adopted a nonlinear modelling framework that includes all three pollutants simultaneously. The case-crossover model is a statistical approach tailored for epidemiological studies to assess the impact of transient exposures on the risk of acute events [4, 14, 16]. The methodology divides time into matched strata, and inference is based on how events are distributed within each stratum rather than on the overall number of events. In this study, a time-stratified referent frame is used, which is designed to include four days, aligning control days with the case day by the same weekday across different weeks [17].

The model specifies the hazard function for an individual *i* on day *t*, given by


$$\begin{array}{c} \lambda_{it}=\Lambda_{it}\;\text{exp}\;\left[\sum_{p=1}^{P}f_{p}(X_{tp})+Z_{t}\right]\\ Z_{t}∼N(0,\sigma_{0}^{2})\\ f_{p}(\cdot )∼{\text{IWP}}_{2}(\sigma_{p}), \end{array}$$

where $\Lambda_{it}$ signifies the baseline hazard. The baseline hazard is assumed to vary smoothly and remain nearly constant within each referent stratum, thereby adjusting automatically for seasonality and day-of-week patterns. The function $f_{p}(X_{tp})$ represents the exposure-response curve for each pollutant and for temperature, and a random effect $Z_{t}∼N(0,\sigma_{0}^{2})$ captures residual day-to-day heterogeneity beyond the mean. Unlike standard Poisson time-series models, this framework imposes only weak assumptions on $\Lambda_{it}$. Although overdispersion is accommodated, we additionally removed major public holidays when healthcare use is atypically low and hospital services may be reduced, to reduce potential bias from irregular care-seeking or limited hospital capacity [18, 19]. We adjusted for temperature because it is the meteorological factor most consistently related to short-term pollution levels and acute cardiorespiratory events in this setting. Other meteorological variables such as humidity, wind speed, and precipitation could also be relevant, but were not included in the main model. We note this as a limitation in the Discussion.

The benefit of the case-crossover model lies in its multinomial partial likelihood function, which does not involve $\Lambda_{it}$, simplifying the model to


$$\pi_{jt}|t\in \Omega_{j}=\frac{\;\text{exp}\;\left[\sum_{p=1}^{P}f_{p}(X_{tp})+Z_{t}\right]}{\sum_{d\in \Omega_{j}}\;\text{exp}\;\left[\sum_{p=1}^{P}f_{p}(X_{dp})+Z_{d}\right]},$$

where $\pi_{jt}$ denotes the conditional probability that an event from stratum *j* (denoted as $\Omega_{j}$) occurs on day *t*, given that one event occurred within that stratum. This within-stratum comparison controls for invariant individual-level confounders and matched calendar effects. For pollutant concentration and temperature, we applied lag effects: the average of lag 0 and lag 1 for pollutants to capture immediate and previous-day effects, and the average of lags 0 to 3 for temperature to capture broader short-term thermal influences. These lag choices are common in studies of this type [4, 5, 20]. Although the model is written in terms of an individual hazard, estimation is based on grouped daily counts. Because exposures are shared within day and stratum, these counts provide sufficient statistics for the corresponding aggregate-data case-crossover analysis.

The exposure-response functions $f_{p}(\cdot )$ are modeled as Gaussian processes, more specifically second-order Integrated Wiener Processes (the continuous counterpart of a second-order random walk; see [21]). This choice provides a flexible penalty on local curvature, allowing nonlinear trends without fixing spline knots in advance. This formulation is closely related to standard spline-smoothing and generalized additive model approaches, with the Gaussian-process representation providing an equivalent way to regularize curvature in the estimated exposure-response function. The standard deviation parameters $\sigma_{p}$ control how rough each function can be and are estimated from the data. We note that an IWP_2_ prior does not imply infinitely smooth sample paths. Rather, it regularizes abrupt changes while permitting realistic roughness in the estimated exposure-response relationship.

Exponential prior distributions were used for all standard deviation parameters, with prior medians of 0.01 for $\sigma_{0}$ and 0.005 for $\sigma_{p}$. These priors favor values near zero, encouraging simpler exposure-response functions with less local curvature, which correspond to linear effects when supported by data. Posterior inference was obtained using adaptive Gauss-Hermite quadrature within a latent Gaussian modelling framework rather than full Markov chain Monte Carlo sampling [17], which is computationally efficient for high-dimensional daily time-series data.

### Two-step implementation algorithm

Our analysis employs a two-step algorithm to systematically evaluate health associations across CDs while accounting for uncertainty in the air pollution exposures, and is based on the air pollution model and method for pooling across CDs from [16]. This approach is integral to our study of the exposure-response relationship, facilitating both CD-specific assessments and comprehensive pooled analysis, enriching our understanding of the environmental impacts on health.

#### Step 1: a bayesian cut model for one CD

For each CD *k*, we begin by constructing a model based on the health data $H_{k}$, aiming to estimate the effects:


$$\left[f_{kp}(x)|H_{k},Y_{k}^{\left[\text{clean}\right]}\right].$$

While health outcome data $H_{k}$ is given, the clean pollution data $Y_{k}^{\left[\text{clean}\right]}$, are not directly observable. We apply a Bayesian integration to exclude $Y_{k}^{\left[\text{clean}\right]}$ through integration. This process results in the full Bayesian model:


$$\left[f_{kp}(x)|H_{k},Y_{k}^{\left[\text{obs}\right]}\right]=\int \left[f_{kp}(x)|H_{k},Y_{k}^{\left[\text{clean}\right]}\right]\left[Y_{k}^{\left[\text{clean}\right]}|H_{k},Y_{k}^{\left[\text{obs}\right]}\right]dY_{k}^{\left[\text{clean}\right]},$$

which is approximated in the cut model (where the conditioning on $H_{k}$ in the second term is ignored) as:


$$\left[f_{kp}(x)|H_{k},Y_{k}^{\left[\text{obs}\right]}\right]\approx \int \left[f_{kp}(x)|H_{k},Y_{k}^{\left[\text{clean}\right]}\right]\left[Y_{k}^{\left[\text{clean}\right]}|Y_{k}^{\left[\text{obs}\right]}\right]dY_{k}^{\left[\text{clean}\right]}.$$

Utilizing the cut model is computationally much simpler than fully-Bayesian inference. It allows for the air pollution model to be run separately and just once, with the output being reusable for multiple analyses.

#### Step 2: Combining health impacts across samples and CDs

Our approach to combining health impacts across different pollution exposure samples and CDs is similar to the methodology of [16]. Specifically we obtain the posterior samples of the risk effect ${\hat{f}}_{kp}^{\left(i\right)}(x)$ for the *k*-th CD and the *i*-th posterior sample, where i=1,…,N, N=S×M, with S=50 representing the number of exposure samples, and M=100 denoting the number of posterior samples from each health model.

The proposed Bayesian hierarchical model used for combining health impacts, for a specific exposure level $x_{0}$ of a single exposure *p*, is defined as follows:


$$\begin{array}{c} {\hat{f}}_{kp}(x_{0})∼N(\beta_{kp},{\hat{\sigma }}_{kp}^{2}),\;k=1,\ldots ,K\\ \beta_{kp}∼N(\beta_{p},\sigma_{p}^{2})\\ \beta_{p}∼N(0,100^{2})\\ \sigma_{p}^{2}∼\text{Inverse-Gamma}(0.001,0.001), \end{array}$$

where


$$\begin{array}{c} {\hat{f}}_{kp}(x_{0})=\frac{\sum_{i=1}^{N}{\hat{f}}_{kp}^{\left(i\right)}(x_{0})}{N}\\ {\hat{\sigma }}_{kp}^{2}=\frac{\sum_{i=1}^{N}{\left({\hat{f}}_{kp}^{\left(i\right)}(x_{0})-{\hat{f}}_{kp}(x_{0})\right)}^{2}}{N-1}. \end{array}$$

Here, $\beta_{kp}$ represents the true effect for the *k*-th CD and $\beta_{p}$ represents the overall effect for the *p*-th pollutant. The priors $\beta_{p}∼N(0,100^{2})$ and $\sigma_{p}^{2}∼\text{Inverse-Gamma}(0.001,0.001)$ are weakly informative, allowing the data to dominate the posterior distributions. The pooling model was fit in Stan via Hamiltonian Monte Carlo using two chains with 15 000 iterations per chain, 10 000 warm-up iterations, thinning every 10 draws. Convergence was assessed using trace plots. This yielded 1000 retained posterior draws for each pooled effect. Together with the 50 posterior exposure samples and 100 posterior samples from each CD-specific health model, this setup propagated uncertainty from both the exposure and health-model stages.

### Assessing joint health impacts of correlated air pollutants

This study also estimated the combined health effect of two correlated pollutants, PM${\;}_{2.5}$ and NO_2_. Due to their strong positive correlation and seasonal consistency, as indicated in Supplementary Table 2, accurately disentangling their independent health impacts was challenging. While the sum of their effects was generally well identified, it was often unclear whether the observed health impacts were attributable primarily to PM${\;}_{2.5}$, to NO_2_, or to both together. For less common health outcomes, the uncertainty around the separate PM${\;}_{2.5}$ and NO_2_ effects was substantial. To address this, we used a data-driven principal component analysis (PCA) to derive a weighted average of the two effects. The weights, 0.325 for PM${\;}_{2.5}$ and 0.675 for NO_2_, were taken from the first principal component coefficients, corresponding to loadings of 0.433 for PM${\;}_{2.5}$ and 0.901 for NO_2_. The first principal component explained 66.8% of the total variability in the two-pollutant data, indicating that it captured the dominant shared temporal variability between these exposures. This combined quantity is not an additional component of the fitted model. Rather, it is a post hoc summary of the estimated PM${\;}_{2.5}$ and NO_2_ effects, used to present a simpler and more stable summary for two highly collinear pollutants while retaining interpretable concentration-response contrasts. Alternative weights based on an air-quality index could also be considered, but the PCA weights were retained here because they reflect the dominant shared variation in these data.

In presenting the results, we use percent change in relative risk, derived from $R_{\%}=100\times [\text{exp}\;\{f(x)\}-1]$, where f(x) denotes the log relative risk for an exposure of *x* units relative to a reference value (10, 20 and 20 for PM${\;}_{2.5}$, NO_2_ and O_3_, respectively). These reference values were chosen to provide interpretable contrasts. We used 10 for PM${\;}_{2.5}$ because this scale is common in the short-term air-pollution literature [4], and 20 for NO_2_ and O_3_ because their concentrations were higher in our data. These choices are somewhat arbitrary and mainly intended to aid interpretation.

## Results

**Figure 2:**
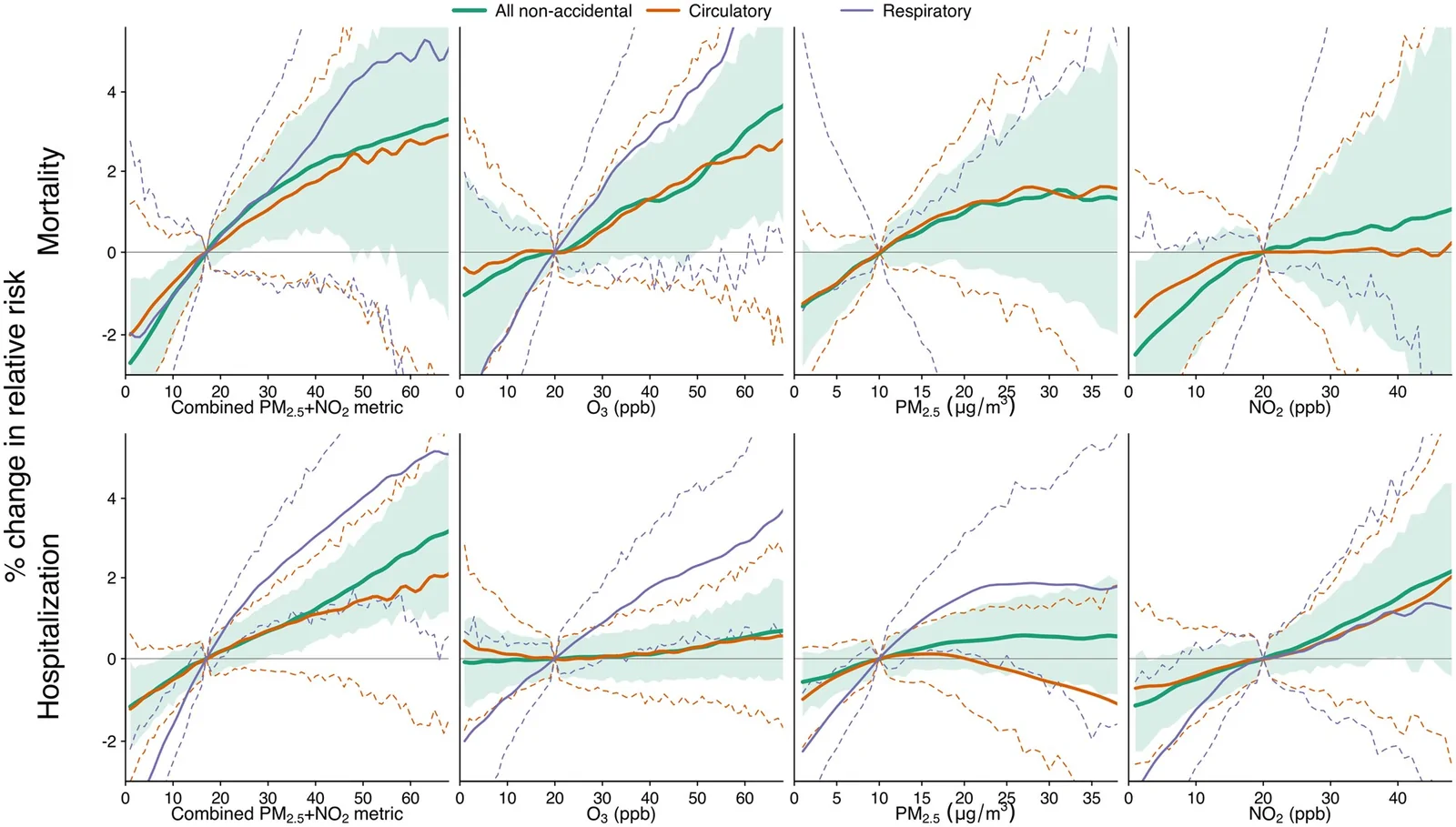
All-age mortality and hospitalization results. Percent change in relative risk is shown for all non-accidental (green), circulatory (orange), and respiratory (purple) outcomes. Solid lines denote posterior means; dashed lines and green shading denote 95% credible intervals. The combined PM${\;}_{2.5}$+NO_2_ metric is a post hoc summary with weights of 0.325 for PM${\;}_{2.5}$ and 0.675 for NO_2_. Respiratory posterior-mean lines are not displayed in the separate PM${\;}_{2.5}$ and NO_2_ mortality panels because the corresponding credible intervals are too wide for reliable interpretation.

**Figure 3:**
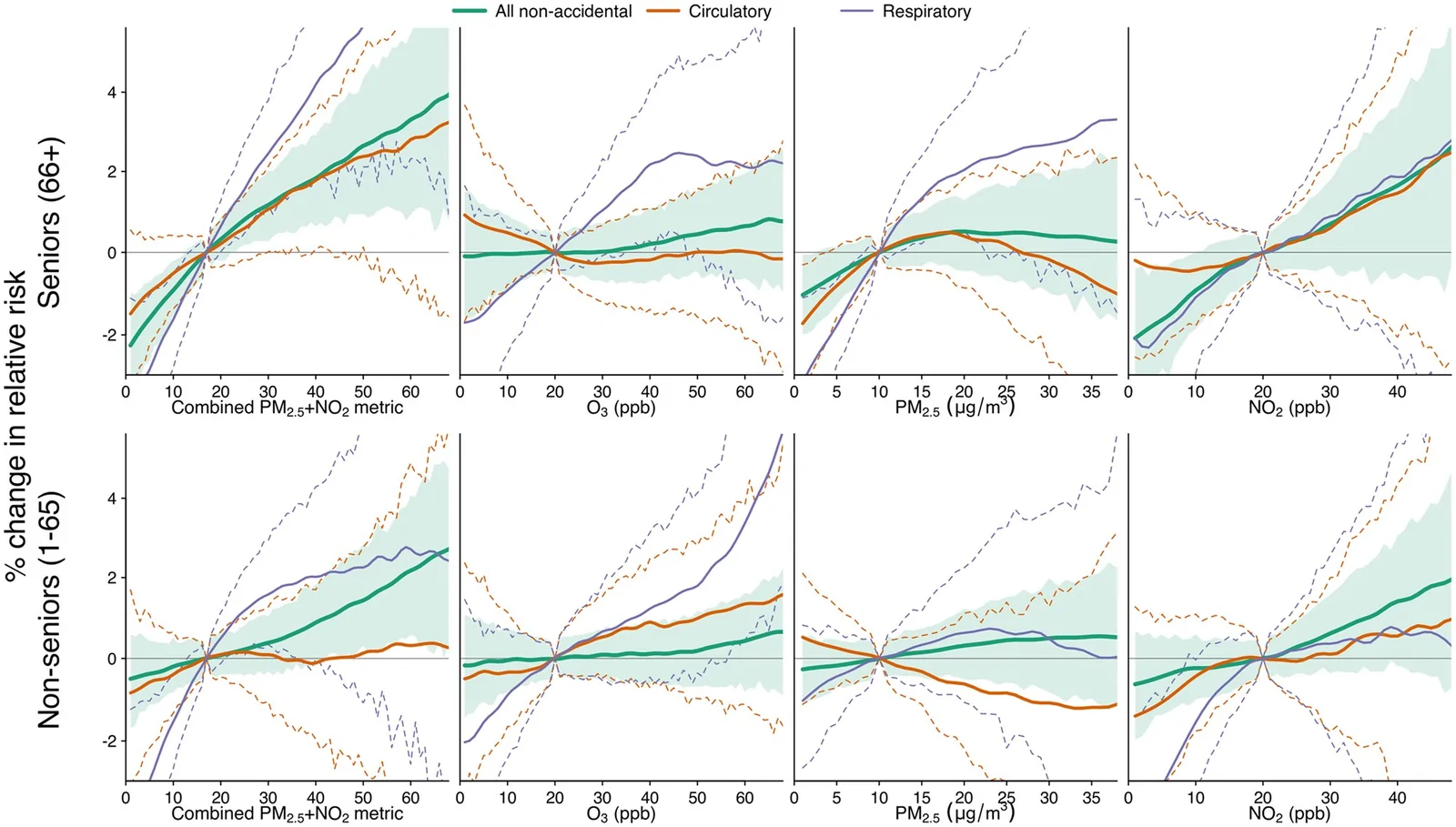
Age-specific hospitalization results. Percent change in relative risk is shown separately for seniors and non-seniors for all non-accidental (green), circulatory (orange), and respiratory (purple) outcomes, across the same pollutant metrics as in Figure 2. Solid lines denote posterior means; dashed lines and green shading denote 95% credible intervals.

Figure 2 illustrates the all-age results for mortality and hospitalization, including the combined PM${\;}_{2.5}$+NO_2_ metric (left column) and the individual effects of each pollutant on three outcomes (all non-accidental, circulatory, and respiratory, shown in different colors). Figure 3 presents hospitalization results for seniors versus non-seniors, and Supplementary Figure 1 shows the corresponding mortality age contrasts.

In Fig. 2, the point estimates are in solid lines and the 95% credible intervals (CrIs) are in dashed lines. The green shaded regions indicate the 95% CrIs for all non-accidental outcomes, showing that the 95% CrIs are narrow for all non-accidental, whereas those for respiratory are quite wide, reflecting the relatively small number of cases (see Table 1).

### Nonlinear mortality impacts of multi-pollutant exposure

The upper panels of Fig. 2 show mortality relative risk across concentrations of combined PM${\;}_{2.5}$+NO_2_, O_3_, PM${\;}_{2.5}$, and NO_2_. The combined metric shows the clearest increasing pattern with less uncertainty than the separate PM${\;}_{2.5}$ and NO_2_ curves, as expected under strong collinearity. O_3_ appears closer to linear, whereas PM${\;}_{2.5}$ and NO_2_ show more curvature at lower concentrations. For cause-specific mortality, especially respiratory mortality, several credible intervals include the null across much of the exposure range, so these curves are suggestive rather than precise evidence of association.

In addition, our PM${\;}_{2.5}$ effects on all non-accidental mortality are broadly consistent with those reported in the global 652-city analysis by [13]. A detailed comparison, including figures and discussion of constraint choices, is provided in Supplementary Figure 2.

To facilitate comparison with existing air-pollution epidemiologic studies, we also report approximate percent changes in relative risk of selected health outcomes associated with a 10-unit increase in exposure, as summarized in Table 2. These values were obtained roughly by extracting representative contrasts from the fitted exposure-response curves in Figs 2 and 3.

**Table 2:** Percent change in relative risk (RR) for a 10-unit (combined PM${\;}_{2.5}$+NO_2_: 3.25 μg/m^3^ + 6.75 ppb; O_3_: 10 ppb) increase in exposure from the reference point, with 95% credible intervals (CrIs).

Outcome	Exposure	% change in RR (95% CrI)
All non-accidental mortality	Combined PM_2.5_ +NO_2_	1.5 (0.3, 2.2)
All non-accidental hospitalization	Combined PM_2.5_ +NO_2_	0.6 (0.0, 1.3)
Senior all non-accidental hospitalization	Combined PM_2.5_ +NO_2_	1.2 (0.4, 2.0)
Respiratory hospitalization	Combined PM_2.5_ +NO_2_	2.0 (0.5, 3.5)
Senior respiratory hospitalization	Combined PM_2.5_ +NO_2_	2.7 (0.9, 4.5)
Respiratory hospitalization	O_3_	0.9 (0.0, 1.8)

*Notes:* Values are approximate and were obtained roughly by extracting representative contrasts from the fitted exposure-response curves. The combined contrast is 3.25 μg/m^3^ PM${\;}_{2.5}$ + 6.75 ppb NO_2_; the ozone contrast is 10 ppb. CrI, credible interval; NO_2_, nitrogen dioxide; O_3_, ozone; PM${\;}_{2.5}$, particulate matter ≤2.5 μm in aerodynamic diameter; ppb, parts per billion; RR, relative risk.

### Hospitalization risks

The lower panels of Fig. 2 show hospitalization results. Credible intervals are generally narrower than for mortality, consistent with the much larger number of hospitalization events. The combined PM${\;}_{2.5}$+NO_2_ metric again shows the clearest monotonic increase, and the strongest slope is seen for respiratory hospitalization. O_3_ and PM${\;}_{2.5}$ are more clearly associated with respiratory hospitalization than with circulatory hospitalization, while NO_2_ shows more similar slopes across hospitalization outcomes. Even so, several cause-specific credible intervals still overlap the null, so the evidence is clearer for the broad hospitalization outcomes than for each individual cause-specific curve.

### Age-specific outcomes

In addition to the all-age analyses, we examined seniors and non-seniors separately. Figure 3 shows that the combined PM${\;}_{2.5}$+NO_2_ association with all non-accidental hospitalization is positive in both age groups. Point estimates suggested a more pronounced pattern for senior respiratory hospitalization. However, uncertainty was substantial and no formal between-age contrast was estimated. For O_3_, both age groups show similar broad patterns for all non-accidental and circulatory hospitalization, whereas respiratory hospitalization rises more clearly at lower O_3_ concentrations.

For NO_2_, the increase in all non-accidental hospitalization is steeper for seniors than for non-seniors at lower concentrations, while circulatory hospitalization shows less consistent age separation. The mortality age contrasts are shown in Supplementary Figure 1 and are more uncertain because mortality counts, especially for respiratory causes in non-seniors, are much smaller.

## Discussion

This study provides an updated national assessment of the short-term health effects of ambient air pollution in Canada using a nonlinear, multi-pollutant modelling framework. By jointly evaluating PM${\;}_{2.5}$, O_3_, and NO_2_, our approach captures the correlated and potentially nonlinear nature of air pollution impacts on mortality and hospitalization. The findings are broadly consistent with the global 652-city analysis by [13], while extending that literature in three ways: by allowing nonlinear pollutant-response functions, by propagating uncertainty from imputed monitoring data, and by comparing broad outcome groups and age strata within one coherent framework.

Our results also illustrate challenges in analyzing PM${\;}_{2.5}$ and NO_2_ together. These pollutants originate from overlapping but distinct sources. By examining them simultaneously, we gain a more comprehensive understanding of exposure. PM${\;}_{2.5}$ reflects regional and secondary particle formation processes, while NO_2_ represents localized combustion and mobility patterns. This comprehensive analysis offers a more complete picture of the factors influencing air pollution exposure. Additionally, the combined metric provides a more summary for two strongly correlated pollutants whose individual effects were often difficult to discern. The combined metric yielded more precise curves and clearer point-estimate patterns, particularly for senior respiratory hospitalization. However, substantial uncertainty and the absence of a formal between-age contrast limit strong conclusions.

Several limitations should be noted. Our uncertainty propagation addresses missing hourly monitoring data but not all sources of exposure error. Exposures and temperature were averaged to the CD-day level, which may induce spatial aggregation error, especially in larger CDs. Temperature was the only meteorological covariate included, while humidity, wind speed, and precipitation may also contribute to short-term confounding. Some cause-specific and age-specific curves, especially for mortality, remained imprecise and should be interpreted cautiously. This uncertainty is itself informative, as it limits strong conclusions about departures from linearity or from the null.

## Conclusion

This nonlinear multi-pollutant Bayesian case-crossover framework provides a practical way to estimate short-term health risks from correlated ambient pollutants while preserving flexible exposure-response shapes. In this Canadian application, the clearest estimated patterns were seen for the combined PM${\;}_{2.5}$+NO_2_ metric, particularly for all non-accidental mortality, all non-accidental hospitalization, and senior respiratory hospitalization. Several estimates remained imprecise. These findings support cautious further evaluation of multi-pollutant risk assessment.

## Ethics approval

This study was exempt from Health Canada Research Ethics Board review under Article 2.2 of the Tri-Council Policy Statement: Ethical Conduct for Research Involving Humans (2022). It used previously collected de-identified administrative data made available through lawful data custodians, involved no participant contact or additional data linkage, and followed applicable access, privacy, and disclosure requirements.

## Supplementary Material

dyag194_Supplementary_Data

## Data Availability

The health outcome data cannot be shared publicly because they are subject to the access and disclosure requirements of their data custodians. Code and exposure data are available on request.
