# Supplementary material for: Short-term health impacts of air pollution in Canada: a nonlinear multi-pollutant Bayesian case-crossover framework
Source: Int J Epidemiol. 2026 Sep 22;55(5):dyag194. doi: 10.1093/ije/dyag194 (PMC13596778; doi:10.1093/ije/dyag194)
Supplement: Supplementary file 1 [file dyag194_supplementary_data.pdf]

Short-term health impacts of air pollution in Canada: A nonlinear multi-pollutant Bayesian case-crossover framework (Supplementary Material)

Guowen Huang[a] Patrick Brown[b,c] Hwashin Hyun Shin[d,e,*]

[a]Department of Statistical and Actuarial Sciences, Western University, London, Ontario, Canada

[b]Centre for Global Health Research, St Michael's Hospital, Toronto, Ontario, Canada

[c]Department of Statistical Sciences, University of Toronto, Toronto, Ontario, Canada

[d]Department of Mathematics and Statistics, Queen's University, Kingston, Ontario, Canada

[e]Environmental Health Science and Research Bureau, Health Canada, Ottawa, Ontario, Canada

[*]Corresponding author: Environmental Health Science and Research Bureau, Health Canada. 269 Laurier Ave. West. Ottawa, ON, K1A 0K9. Hwashin.Shin@hc-sc.gc.ca

S1 CD-specific total cases

Table S1. Daily average cases for mortality (2001-2015) and hospitalization (2001-2018) by type and age-group (S: senior with age 66+; NS: nonsenior with age 1-65) in each CD.

	**Allcause**				**Circulatory**				**Respiratory**			
	**Mortality**		**Hosp.**		**Mortality**		**Hosp.**		**Mortality**		**Hosp.**	
CD	S	NS	S	NS	S	NS	S	NS	S	NS	S	NS
Halifax	5.3	1.5	19.8	41.4	1.7	0.4	4.9	3.5	0.6	0.1	2.5	2.9
Saint John	1.7	0.4	7.1	10.2	0.7	0.1	1.8	1.0	0.2	0.0	1.1	1.0
Ottawa	10.7	2.8	46.6	81.4	3.6	0.6	11.4	6.5	1.0	0.1	5.9	4.8
Durham	6.6	2.0	32.8	65.2	2.1	0.4	9.0	6.3	0.7	0.1	4.1	4.4
Toronto	34.0	8.4	170.2	264.4	11.2	1.8	44.6	22.9	3.3	0.3	20.8	16.8
Peel	9.2	3.2	55.4	133.2	2.9	0.7	14.8	11.7	0.9	0.1	6.6	9.4
Halton	5.6	1.3	31.0	49.9	1.7	0.2	8.0	4.4	0.5	0.0	3.5	3.0
Hamilton	8.6	2.2	42.3	60.3	2.9	0.5	11.6	6.1	0.9	0.1	5.2	4.1
Niagara	8.6	1.9	39.6	51.4	3.2	0.4	11.3	5.6	0.7	0.1	4.5	4.3
Waterloo	6.2	1.6	26.3	53.3	2.2	0.4	6.8	4.3	0.6	0.1	3.4	3.8
Essex	6.1	1.6	30.4	46.8	2.4	0.4	8.2	4.5	0.5	0.1	3.7	3.2
Lambton	2.5	0.6	12.9	16.0	1.0	0.2	3.5	1.7	0.2	0.0	1.5	1.5
Sudbury	2.8	0.8	15.0	20.7	1.0	0.2	3.9	2.2	0.3	0.0	1.8	1.5
Algoma	2.5	0.6	15.2	18.0	0.9	0.2	4.4	2.4	0.3	0.0	1.8	1.3
Winnipeg	11.3	2.8	42.0	73.8	4.0	0.7	11.1	5.6	1.1	0.1	5.6	4.7
Regina	3.8	1.0	20.2	38.0	1.3	0.2	4.8	2.6	0.4	0.1	2.8	3.9
Saskatoon	3.9	1.0	18.6	35.0	1.4	0.2	4.8	2.5	0.4	0.0	2.3	2.6
Calgary	11.4	3.5	62.6	156.5	4.5	0.8	14.8	10.3	1.1	0.1	7.8	8.5
Edmonton	12.7	4.1	64.4	145.1	4.7	1.0	14.0	9.1	1.4	0.3	9.3	10.7
Vancouver	27.9	6.9	139.8	238.8	10.0	1.4	33.8	16.9	3.1	0.3	17.1	15.0

S2 Correlation among pollutants

Table S2. Correlation among pollutants. The correlation between $PM_{2.5}$ and NO_2 remains consistent in both summer and winter, and their correlations with O_3 are positive in summer and negative in winter.

	**cor(pm25, no2)**			**cor(pm25, o3)**			**cor(no2, o3)**		
CD	allyear	summer	winter	allyear	summer	winter	allyear	summer	winter
Halifax	-0.03	0.05	-0.17	0.24	0.48	0.20	-0.28	-0.17	-0.60
Saint John	0.38	0.45	0.27	0.13	0.42	-0.22	-0.03	0.08	-0.35
Ottawa	0.45	0.54	0.71	0.22	0.61	-0.49	-0.25	0.28	-0.66
Durham	0.46	0.54	0.58	0.32	0.72	-0.46	-0.20	0.38	-0.70
Toronto	0.41	0.53	0.43	0.38	0.67	-0.36	-0.19	0.28	-0.61
Peel	0.40	0.45	0.51	0.31	0.70	-0.41	-0.33	0.11	-0.70
Halton	0.41	0.49	0.47	0.36	0.70	-0.37	-0.27	0.23	-0.71
Hamilton	0.38	0.38	0.48	0.31	0.63	-0.35	-0.37	-0.11	-0.70
Niagara	0.23	0.31	0.42	0.48	0.69	-0.17	-0.23	0.16	-0.55
Waterloo	0.30	0.36	0.47	0.42	0.70	-0.19	-0.19	0.32	-0.42
Essex	0.28	0.44	0.38	0.37	0.64	-0.27	-0.40	0.08	-0.57
Lambton	0.53	0.59	0.55	0.24	0.66	-0.45	-0.23	0.35	-0.73
Sudbury	0.30	0.61	0.57	0.36	0.67	-0.44	-0.13	0.34	-0.67
Algoma	0.30	0.44	0.56	0.42	0.68	-0.28	-0.04	0.27	-0.38
Winnipeg	0.12	0.05	0.42	0.25	0.39	-0.22	-0.15	0.11	-0.47
Regina	0.03	0.16	0.23	0.12	0.26	-0.40	-0.22	-0.12	-0.25
Saskatoon	0.02	0.11	0.38	0.19	0.21	-0.20	-0.23	-0.04	-0.26
Calgary	0.09	0.20	0.44	0.00	0.24	-0.61	-0.57	0.04	-0.67
Edmonton	0.22	0.12	0.47	-0.04	0.22	-0.54	-0.53	0.03	-0.75
Vancouver	0.37	0.48	0.59	-0.07	0.40	-0.57	-0.48	0.32	-0.66

S3 CD-specific exposure summaries

Table S3. A summary of CD-specific exposures to $PM_{2.5}$ (daily mean in µg/m^3), NO_2 (daily mean in ppb), O_3 (daily 8-hour maximum in ppb), and temperature (daily mean in°C) from 2001 to 2018.

	**$PM_{2.5}$**				**NO_2**				**O_3**				**Temperature**			
CD	med	q10	q90	q99	med	q10	q90	q99	med	q10	q90	q99	med	q10	q90	q99
Halifax	6.1	3.3	9.4	15.6	10.2	4.4	18.3	31.6	26.5	17.6	36.0	42.8	8.2	-3.6	18.9	21.9
Saint John	6.2	2.8	10.1	20.0	5.3	2.1	9.6	15.7	29.8	19.7	40.0	47.7	6.7	-6.2	17.1	20.0
Ottawa	6.3	1.9	12.3	26.2	8.8	3.1	16.8	28.5	32.1	18.5	46.2	61.0	7.9	-8.8	21.7	26.1
Durham	7.4	2.4	14.1	30.1	9.4	4.6	15.6	24.3	32.6	19.5	45.9	61.9	8.4	-6.1	21.0	25.0
Toronto	8.0	2.7	15.1	31.6	17.9	9.2	28.5	41.4	31.4	16.4	47.6	67.5	10.0	-4.0	22.6	26.6
Peel	7.7	2.5	14.7	30.0	13.2	5.4	22.7	34.2	34.8	18.9	50.5	69.7	9.6	-5.2	22.7	27.4
Halton	7.8	2.5	14.7	30.3	11.9	5.7	19.8	29.9	34.0	19.1	49.0	67.3	9.9	-4.1	22.4	26.9
Hamilton	8.6	2.9	16.3	30.8	13.3	6.1	21.9	32.5	33.5	18.5	49.4	68.1	9.2	-5.1	21.8	26.2
Niagara	7.7	2.4	14.4	30.2	10.1	4.3	17.4	29.2	36.7	21.3	54.7	74.0	10.2	-3.7	22.6	26.5
Waterloo	7.6	2.3	14.7	29.7	8.8	3.1	16.5	28.4	37.5	22.3	54.6	75.2	8.3	-6.5	21.0	25.3
Essex	8.9	3.2	16.1	30.2	11.8	6.0	18.8	27.6	38.8	19.2	58.8	79.2	10.9	-3.2	23.5	27.2
Lambton	8.7	3.9	15.0	27.0	10.6	4.8	17.6	28.3	37.6	22.6	52.0	67.2	9.7	-4.7	22.4	26.6
Sudbury	6.0	1.9	11.2	23.1	7.1	3.1	12.3	22.7	33.2	21.0	46.5	62.0	5.4	-11.9	19.8	24.2
Algoma	7.2	2.1	14.0	25.0	8.8	3.6	16.0	25.3	33.4	21.8	46.1	60.9	4.8	-11.3	18.1	22.2
Winnipeg	5.7	2.1	9.9	19.7	9.4	3.4	17.4	27.7	28.6	15.6	42.4	53.4	5.7	-14.1	21.7	26.4
Regina	7.7	3.0	13.0	25.1	10.6	5.1	17.4	26.7	27.6	16.5	41.1	50.6	4.0	-14.8	18.9	23.6
Saskatoon	6.4	1.8	11.5	23.2	10.1	4.4	17.3	26.8	29.4	16.5	43.1	53.3	4.0	-14.6	18.9	23.4
Calgary	7.6	3.2	12.5	28.1	16.2	6.7	27.7	38.3	31.8	15.2	48.5	58.7	4.8	-8.8	16.6	20.7
Edmonton	8.0	3.1	13.7	32.2	14.4	6.1	26.1	38.3	29.5	12.7	46.2	57.3	4.5	-11.1	17.3	21.6
Vancouver	6.0	2.5	10.1	18.3	15.3	9.0	22.5	30.5	21.9	8.2	34.3	41.4	10.8	3.4	18.6	22.5

Note: 'med' refers to the median, 'q10, q90, q99' indicates the 10th, 90th, 99th quantile, respectively.

S4 Age-specific health effects

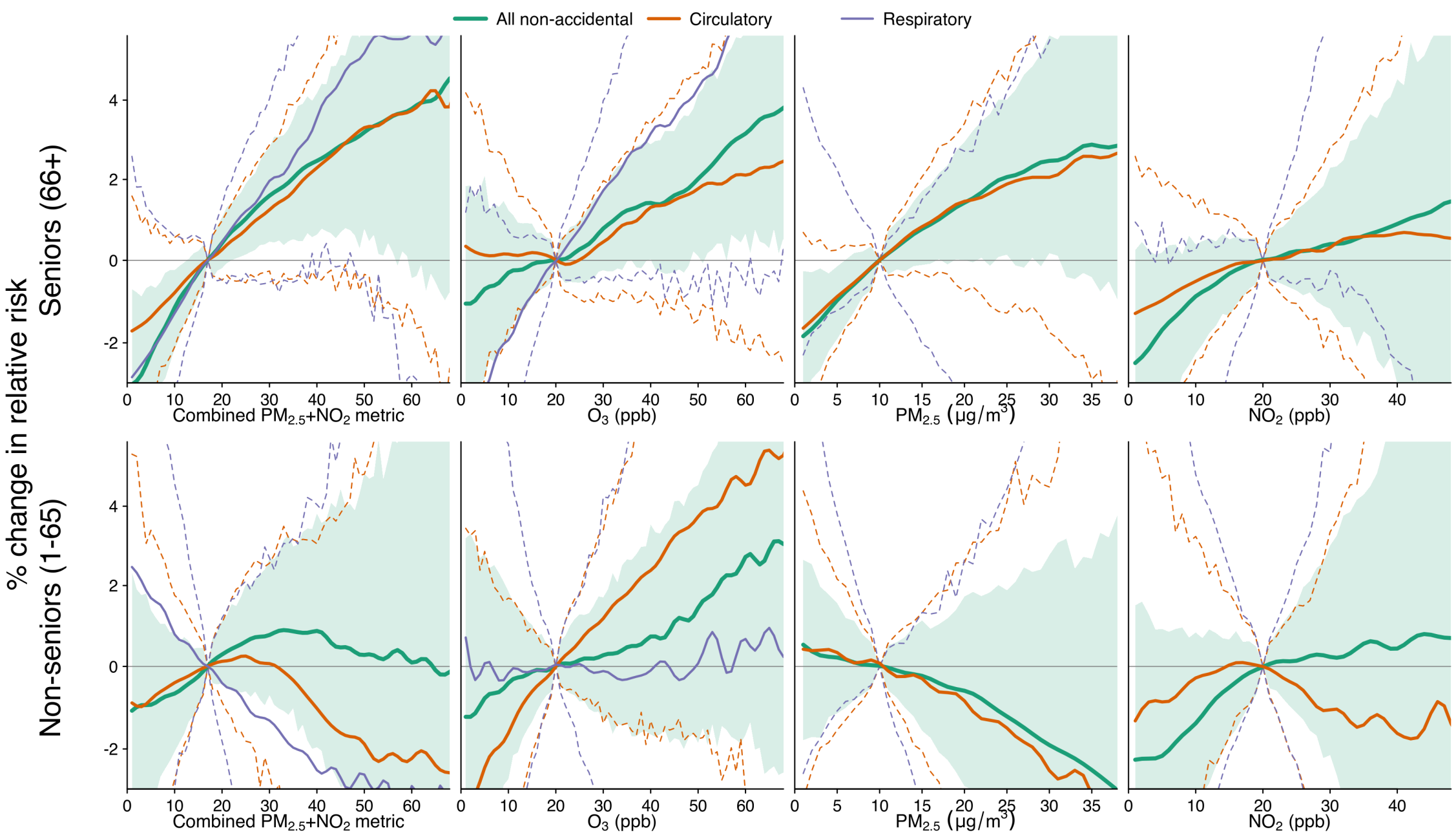

Figure S1. Age-specific mortality results. Percent change in relative risk is shown separately for seniors and non-seniors for all non-accidental (green), circulatory (orange), and respiratory (purple) outcomes across the combined $PM_{2.5}$+NO_2 metric, O_3, $PM_{2.5}$, and NO_2. Solid lines denote posterior means; dashed lines and green shading denote 95% credible intervals. The combined $PM_{2.5}$+NO_2 metric is a post hoc summary with weights of 0.325 for $PM_{2.5}$ and 0.675 for NO_2. Respiratory posterior-mean lines are not displayed in the separate $PM_{2.5}$ and NO_2 panels because the corresponding credible intervals are too wide for reliable interpretation.

Alt text: Eight-panel plot comparing mortality exposure-response curves for seniors and non-seniors across the combined $PM_{2.5}$+NO_2 metric, O_3, $PM_{2.5}$, and NO_2. Green shading and orange and purple dashed lines show 95% credible intervals. Respiratory posterior-mean lines are not displayed in the separate $PM_{2.5}$ and NO_2 panels because the corresponding credible intervals are too wide for reliable interpretation.

S5 Comparison with a global 652-city study

Supplementary Figure S2 presents a comparative analysis of the impact of $PM_{2.5}$ on all non-accidental mortality between the present study (2001-2015) and the research conducted by Liu et al. [1] (1986-2011), which investigated the association between ambient particulate air pollution and daily mortality across 652 cities. To facilitate direct comparison, the present study's results are

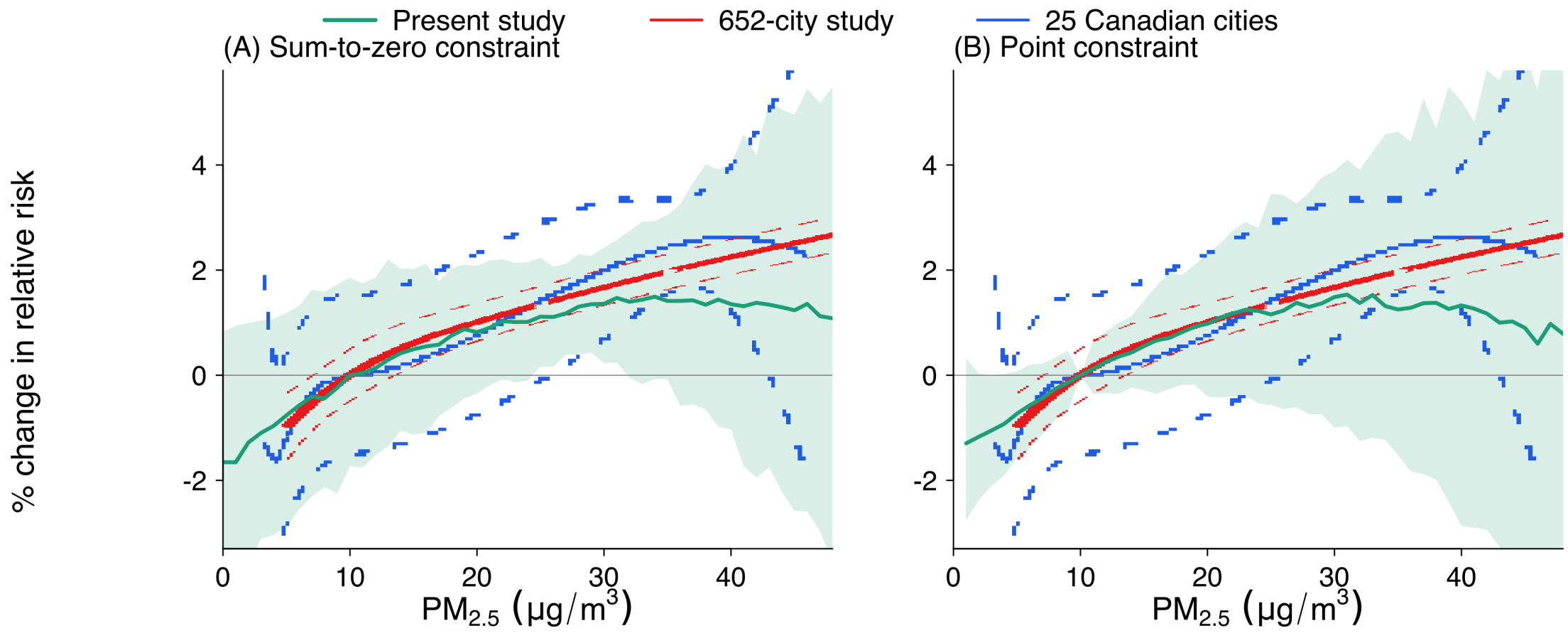

Figure S2. Estimated $PM_{2.5}$ associations with all non-accidental mortality in the present study (green) and those reported by Liu et al. [1] for 652 cities (red) and 25 Canadian cities (blue), under (A) sum-to-zero and (B) point constraints. Solid lines are central estimates; dashed red and blue lines and green shading denote the reported uncertainty intervals.

Alt text: Two side-by-side $PM_{2.5}$ exposure-response plots compare the present study with estimates from Liu et al. for 652 cities and 25 Canadian cities under sum-to-zero and point constraints. Curves are broadly similar at lower concentrations, with uncertainty varying at the extremes of the exposure range.

displayed under both sum-to-zero and point constraints, shown in panel A and panel B, respectively. The point constraint reflects the original findings of the present study, utilizing a reference point of value 10, whereas the sum-to-zero constraint represents an alternate expression of the original findings, adjusting the aggregate effect across the range ($PM_{2.5}$ concentration) of 10 to 40 to sum to zero.

From Supplementary Figure S2, it is observed that the green line (the present paper) and the red dashed line from Liu et al. [1] for 652 cities track closely together at low levels of $PM_{2.5}$, suggesting that the results from this paper are in general agreement with the findings of Liu et al. [1] for a large number of global cities. Given that our study focuses on Canadian CDs, we correlate our findings with the outcomes represented by the blue lines reported by Liu et al. [1]. The blue solid line, representing the effects in 25 Canadian cities, exhibits a trend that closely aligns with our observations.

Importantly, the blue dashed line (95% CI) demonstrates that the estimations for $PM_{2.5}$ levels between 35 and 40 were achieved with greater precision, as indicated by the narrower confidence intervals in this interval. This increased accuracy may be due to the deliberate placement of knots within the spline functions employed by Liu et al. [1]. This rationale is reinforced by the fact that data points for high $PM_{2.5}$ levels are expected to be rare, as detailed in their paper appendix, which noted that the mean $PM_{2.5}$ level in Canada was 9.3 $\mu g/m^3$ with a standard deviation of 6 $\mu g/m^3$. Correspondingly, our estimates also exhibit a narrower credible interval around the $PM_{2.5}$ value of 30 (see panel A of Supplementary Figure S2), which is partly a consequence of our choice to implement a sum-to-zero constraint across the 10-40 range.

Another key observation from the comparison is the significant uncertainty in the estimated effects at low $PM_{2.5}$ levels in their study, whereas our study offers substantially more precise estimations. This precision is crucial given that most of Canada's air pollution levels fall within this low $PM_{2.5}$ range. The significant uncertainty in their estimates is likely due to how the knots are positioned in the spline functions in their model. This underscores the strength of our study, which enhances the exposure-response function through the use of the Integrated Wiener Processes (IWP) model.

References

1. Liu C, Chen R, Sera F *et al.* Ambient particulate air pollution and daily mortality in 652 cities. *N Engl J Med* 2019;**381**:705–15. https://doi.org/10.1056/NEJMoa1817364.
